# β‐Catenin in desmoid‐type fibromatosis: deep insights into the role of T41A and S45F mutations on protein structure and gene expression

**DOI:** 10.1002/1878-0261.12101

**Published:** 2017-09-29

**Authors:** Chiara Colombo, Antonino Belfiore, Nicholas Paielli, Loris De Cecco, Silvana Canevari, Erik Laurini, Maurizio Fermeglia, Sabrina Pricl, Paolo Verderio, Stefano Bottelli, Marco Fiore, Silvia Stacchiotti, Elena Palassini, Alessandro Gronchi, Silvana Pilotti, Federica Perrone

**Affiliations:** ^1^ Sarcoma Service Department of Surgery Fondazione IRCCS Istituto Nazionale Tumori Milan Italy; ^2^ Laboratory of Experimental Molecular Pathology Department of Pathology Fondazione IRCCS Istituto Nazionale Tumori Milan Italy; ^3^ Functional Genomics and Bioinformatics Department of Applied Research and Technology Development Fondazione IRCCS Istituto Nazionale dei Tumori Milan Italy; ^4^ Molecular Simulation Engineering (MOSE) Laboratory DEA University of Trieste Italy; ^5^ Unit of Medical Statistics, Biometry and Bioinformatics Fondazione IRCCS Istituto Nazionale Tumori Milan Italy; ^6^ Adult Mesenchymal Tumor Medical Oncology Unit Fondazione IRCCS Istituto Nazionale Tumori Milan Italy

**Keywords:** desmoid‐type fibromatosis, gene expression, modeling, β‐catenin mutation

## Abstract

Desmoid‐type fibromatosis (DF) is a rare mesenchymal lesion with high risk of local recurrence. Specific β‐catenin mutations (S45F) appeared to be related to this higher risk compared to T41A‐mutated or wild‐type (WT). We explored the influence of both mutations and WT on structure stability and affinity of β‐catenin for α‐catenin and the pattern of gene expression that may influence DF behavior. Using 33 surgically resected primary DFs harboring T41A (*n* = 14), S45F (*n* = 10), or WT (*n* = 9), we performed a comparative molecular analysis by protein/protein interaction modeling, gene expression by DASL microarrays, human inflammation gene panel, and assessment of immune system‐based biomarkers by immunohistochemistry. Mutated proteins were more stable than WT and formed a weaker complex with α‐catenin. Consensus unsupervised gene clustering revealed the presence of two DF group‐mutated (T41A + S45F) and WT (*P *=* *0.0047). The gene sets ‘Inflammatory‐Defense‐Humoral Immune Response’ and **‘**Antigen Binding’ were significantly enriched in T41A. The deregulation of 16 inflammation‐related genes was confirmed. Low numbers of T cells and tumor‐associated macrophages (TAM) infiltrating the tumors and low/absent PD‐1/PD‐L1 expression were also identified. We demonstrated that mutated DFs (T41A or S45F) and WT are two distinct molecular subgroups with regard to β‐catenin stability, α‐catenin affinity, and gene expression profiling. A different inflammation signature characterized the two mutated groups, suggesting mediation either by T41A or by S45F. Finally, all mutated cases showed a low number of TIL and TAM cells and a low or absent expression of PD‐1 and PD‐L1 consistent with β‐catenin activation insensitive to checkpoint blockade.

**Abbreviations**
ADL: average dynamic length
DF: desmoid‐type fibromatosis
FDR: false discovery rate
FFPE: formalin‐fixed paraffin‐embedded
GSEA: gene set enrichment analysis
MD: molecular dynamics
NES: normalized enrichment score
n: number
PAR: progression arrest rate
RFS: recurrence‐free survival
TAM: tumor‐associated macrophages
TIL: tumor‐infiltrating lymphocytes
WT: wild‐type

## 1 Introduction

Desmoid‐type fibromatosis (DF) is a rare mesenchymal infiltrative lesion that exhibits high risk of local recurrence despite surgery with negative resection margins. The high propensity to relapse, the need for multiple surgeries with consequent functional and cosmetic impairment, the observation of prolonged stability in the absence of specific treatments, and the description of spontaneous regression have let the sarcoma community to change the management of this disease toward a more conservative tendency (Bonvalot *et al*., 2008; Fiore *et al*., 2009). Recently, guidelines have underlined the possibility of proposing a frontline observational approach to almost all patients with newly DF reserving specific treatments such as different drug regimens, radiotherapy, or surgery to selected cases including progressive disease (Kasper *et al*., 2015). Unfortunately, changing in the clinical practice has not been paralleled by increasing knowledge regarding the pathogenesis and progression of this rare disease. Accurate molecular predictors that can be used at the beginning of the clinical history to tailor specific treatments are not available to date. Mutations in *CTNNB1* gene encoding for β‐catenin are typically carried by most sporadic DFs. Three different β‐catenin point mutations, T41A, S45F, and S45P, have been described in the vast majority of DFs and have been associated with the prevention of protein phosphorylation and subsequent protein stabilization with translocation to the nucleus, where β‐catenin regulates transcription through its interaction with TCF/LEF (Kundu *et al*., 2006). These β‐catenin mutations were evaluated for their impact on DF patients’ outcome. By comparing mutated and wild‐type (WT) surgical DFs, we and other groups observed a statistically significant association between *CTNNB1* mutations and higher risk for local recurrence (van Broekhoven *et al*., 2015; Colombo *et al*., 2013; Dômont *et al*., 2010). In particular, the specific mutation S45F was associated with worse recurrence‐free survival (RFS) (van Broekhoven *et al*., 2015; Colombo *et al*., 2013; Lazar *et al*., 2008). However, this evidence was not confirmed by other groups who reported a statistically not significant trend of poorer outcome in patients harboring the S45F mutation (Dômont *et al*., 2010) or no correlation between the specific *CTNNB1* mutation and RFS (Mullen *et al*., 2013; Romero *et al*., 2012). Regarding the impact of mutations on response to medical treatment, Nishida *et al*. did not find a correlation between the efficacy of methotrexate or vinblastine and specific *CTNNB1* mutations (Nishida *et al*., 2015). On the other hand, only the S45F mutation was significantly associated with a poor response to meloxicam (Hamada *et al*., 2016) and with a progression arrest rate after imatinib treatment (Kasper *et al*., 2016).

In this scenario of contrasting evidence of the impact of *CTNNB1* mutations and considering that these mutations map in the N‐terminal domain of β‐catenin that mediates both its degradation and interaction with α‐catenin in the cell–cell adhesions (Jiang and Struhl, 1998; Pokutta *et al*., 2014), we hypothesized that the mutation type could influence the stability of the β‐catenin structure, its affinity for α‐catenin, as well as the pattern of gene expression that in turn may guide the behavior of the disease. Thus, in this work, we performed a comparative molecular analysis of protein structure based on protein/protein interaction modeling and of gene and protein expression.

## 2 Materials and methods

### 2.1 Patients and samples

For this study, we selected 33 primary DFs surgically treated at our institution. All samples had the following characteristics: (a) DF diagnosis by an expert pathologist (SP); (b) known *CTNNB1* mutational status assessed by direct sequencing as previously described (Colombo *et al*., 2013); (c) availability of the formalin‐fixed paraffin‐embedded (FFPE) surgical specimen; (d) availability of RNA amount and quality adequate for molecular analysis. The distribution of mutations was T41A (14 cases), S45F (10 cases), and WT (nine cases). Patients and disease characteristics are detailed in Table 1. Data retrieved were gender, age at surgery, anatomical site (abdominal wall, intra‐abdominal, and extra‐abdominal including extremity/girdle, head/neck, thoracic wall), size (cm), *CTNNB1* mutational status, data of surgery, data of recurrence, data of last follow‐up, status at last follow‐up. All patients had a macroscopically complete (including R0 and R1 margins) surgical resection except one. All samples were obtained after informed consent from patients. The study was approved by the Independent Ethics Committee of Fondazione IRCCS Istituto Nazionale dei Tumori (Approval Number RF‐2009‐1511297).

**Table 1:** Patients and disease characteristics

No.	Age (years)	Anatomical site	Size (cm)	CTNNB1 status	Recurrence	Margins	Adjuvant RT	Status at last follow‐up
1	60	Extra‐abdominal	4	T41A	NO	R1	Yes	NED
2	39	Abdominal wall	5	WT	NO	R0	No	NED
3	36	Abdominal wall	15	S45F	NO	R0	No	NED
4	32	Abdominal wall	5	T41A	NO	R0	No	NED
5	53	Extra‐abdominal	12	S45F	15/03/2007	R0	Yes	AWD
6	49	Intra‐abdominal	4	T41A	NO	R0	No	NED
7	16	Extra‐abdominal	3	WT	NO	R0	No	NED
8	26	Extra‐abdominal	2	WT	NO	R1	No	NED
9	19	Abdominal wall	10	S45F	NO	R0	No	NED
10	33	Extra‐abdominal	5	T41A	NO	R1	No	NED
11	67	Extra‐abdominal	4	WT	NO	R0	No	NED
12	42	Abdominal wall	6	S45F	NO	R0	No	NED
13	32	Abdominal wall	8	S45F	NO	R0	No	NED
14	25	Extra‐abdominal	7	T41A	NO	R1	No	NED
15	69	Intra‐abdominal	4	T41A	NO	R0	No	NED
16	39	Abdominal wall	6	WT	NO	R1	No	NED
17	70	Extra‐abdominal	6	WT	NO	R0	No	NED
18	32	Extra‐abdominal	5	T41A	YES	R1	No	NED
19	32	Abdominal wall	6	T41A	NO	R0	No	NED
20	17	Extra‐abdominal	9	T41A	NO	R0	No	NED
21	28	Intra‐abdominal	18	T41A	NO	R0	No	NED
22	33	Abdominal wall	10	S45F	NO	R0	No	NED
23	36	Extra‐abdominal	5	WT	15/07/2006	R0	No	AWD
24	51	Extra‐abdominal	–	T41A	NO	R1	No	NED
25	37	Intra‐abdominal	28	WT	NO	R0	No	NED
26	25	Abdominal wall	15	WT	NO	R0	No	NED
27	47	Extra‐abdominal	11	S45F	NO	R1	Yes	NED
28	31	Intra‐abdominal	–	T41A	NO	R2	Yes	AWD
29	45	Intra‐abdominal	3	T41A	NO	R0	No	DOC
30	43	Abdominal wall	7	T41A	NO	R0	No	NED
31	50	Extra‐abdominal	7	S45F	NO	R0	No	NED
32	43	Extra‐abdominal	11	S45F	3/05/2007	R1	No	AWD
33	24	Extra‐abdominal	6	S45F	5/10/2005	R0	No	AWD

NED, not evidence of disease; AWD, alive with disease; DOC, dead for other cause.

### 2.2 Modeling

The optimized full‐length three‐dimensional (3D) models of WT and mutant isoforms of β‐catenin were obtained by a combination of homology modeling techniques with molecular dynamics (MD) refinements (Bozzi *et al*., 2013; Dileo *et al*., 2011; Laurini *et al*., 2011; Morgan *et al*., 2015). WT and mutant β‐catenin thermodynamic stability was determined by MD simulations in the molecular mechanics/Poisson Boltzmann surface area (MM/PBSA) framework of theory (Ferrone *et al*., 2006; Gibbons *et al*., 2014; Negri *et al*., 2009; Pierotti *et al*., 2010, 2011; Pricl *et al*., 2015). The available structure of β‐catenin (4IGG pdb) was used to build all WT and mutant β‐catenin/α‐catenin protein–protein complex and to determine the dynamics and energetics of their interactions via MM/PBSA MD simulations (see Data S1 for details).

### 2.3 RNA isolation and whole‐genome gene expression profiling

The sample areas containing more than 90% tumor cells without necrosis or surrounding normal tissue were chosen for the analysis and macrodissected. Total RNA was isolated using Qiagen RNeasy FFPE Kit (Qiagen, Valencia, CA, USA) following the manufacturer's instructions and quantified by Nanodrop‐1000 instrument (Thermo Fisher Scientific, Waltham, MA, USA). RNA quality was assessed by RT‐qPCR amplifying amplicons of different size for ACTB housekeeping gene (Mittempergher *et al*., 2011; Ravo *et al*., 2008). Two hundred nanogram of RNA from each sample was subjected to profiling on Human WG‐DASL Assay with Human HT12 v4.0 BeadChips (Illumina Inc., San Diego, CA, USA) that allows the expression profiling of 29 377 probes from degraded RNA, such as that extracted from FFPE samples (Vallacchi *et al*., 2014). Reverse transcription, oligo annealing, ligation, amplification, labeling, probe purification, hybridization, and chip's washing were performed following the manufacturer's instructions. Microarray chips were scanned with an Illumina BeadArray Reader. Primary data were extracted by genome studio software V.3 (Illumina Inc.), normalized by quantile algorithm, and exported without correcting for background or scale. All microarray data are MIAME compliant and the raw data were deposited into the NCBI's Gene Expression Omnibus (GEO) database (http://www.ncbi.nmlm.nih.gov/projects/geo/) with accession number GSE65021

### 2.4 Statistical and bioinformatic analyses

Statistical analysis was performed using R Development Core Team, 2007 version 2.15, BioConductor (Available online: www.bioconductor.org) and the BRB‐ArrayTool developed by Richard Simon and the BRB‐ArrayTools Development Team (v4.2.0; National Cancer Institute, USA). Unsupervised tumor subtype identification was performed by ConsensusClusterPlus (Wilkerson and Hayes, 2010). Supervised class comparison was made to define the differentially expressed genes imposing a significance threshold of false discovery rate (FDR) < 1% and |log2 (fold change)| > 1 between WT and mutated *CTNNB1*‐bearing samples. The probability of finding probe sets significant by chance (FDR at 0.01 level) was determined by the global test through 1000 permutations as computed by BRB tool using default parameters. Pathway analysis on 905 GO terms was performed using gene set enrichment analysis (GSEA). The degree of gene sets’ enrichment in the WT and mutated DF was represented by a normalized enrichment score (NES) and NES with FDR < 0.05 and > |1.5| was considered statistically significant. Graphical representation of GSEA findings was provided by GOBubble plot function of GOplot R package (Wencke *et al*., 2015), displaying information about the significance of the enrichment (−log10‐adjusted *P*‐value) and the z‐score of each gene set. For comparison purposes, the gene list derived from 14 sporadic DF (not characterized for *CNTBB1* mutation) and five paired normal samples plus six solitary fibrous tumor specimens was analyzed (Colombo *et al*., 2011). The list of 636 and 119 genes, up and down (see Table S2 in Colombo *et al*., 2011) in DF, respectively, was imported in GSEA and used as custom gene sets and tested for enrichment in our gene expression dataset.

### 2.5 Nanostring nCounter System

The Nanostring nCounter System analysis was performed by Pharma Diagen (Padova, Italy) using the Human Inflammation panel (249 genes). Statistical analysis was conducted by comparing WT versus both mutated cases and then the mutation T41A versus S45F. Raw data from Nanostring platform were normalized by resorting to a two‐step strategy using the R NanoStringNorm package (Waggott *et al*., 2012). Firstly, by taking advantage of the negative and positive controls present in the Nanostring assay, a technical normalization was performed. A biological normalization was then implemented by using the housekeeping genes as endogenous controls. Subsequently, due to the limited sample size per group, we considered only that genes resulting expressed in all samples for the comparisons of interest. To analyze the normalized gene copies counts, a generalized linear model was implemented with a logarithmic link that takes into account overdispersion of the data (Agresti, 2007). Multiple testing adjustment, such as FDR correction (at 0.05 level), was used to manage the multiplicity problem. Statistical analyses were performed with r and sas software (version 9.2.; SAS Institute Inc., Cary, NC, USA).

### 2.6 Immunohistochemical analysis

Immunohistochemistry was performed on 2‐μm FFPE sections in an automated immunostainer (Autostainer Link48; Dako, Glostrup, Denmark) according to the manufacturer's instructions. The following antibodies were used: β‐catenin 14 (1 : 1000; BD Transduction, South San Francisco, CA, USA), CD3 polyclonal (1 : 400; Dako), CD4 4B12 (1 : 300; Dako), CD8 C8/144B (1 : 20; Dako), CD20 L26 (1 : 400; Dako), CD21 1F8 (1 : 50; Dako), CD56 123C3 (1 : 400; Dako), CD68 KP1 (1 : 3000; Dako), CD163 10D6 (1 : 200; Novocastra, Newcastle, UK), FOXP3 259D/C7 (1 : 200; BD Pharmingen), PD‐L1 SP142 (1 : 30; SPRING, Pleasanton, CA, USA), PD1 NAT105 (1 : 100; Biocare Medical, Pikenine, Concord, NC, USA), and EZH2 5246S (1 : 50; Cell Signaling, Leiden, Netherlands).

## 3 Results

### 3.1 *CTNNB1*‐mutated and WT DFs have different thermodynamic stability and affinity for α‐catenin

**Figure 1:**
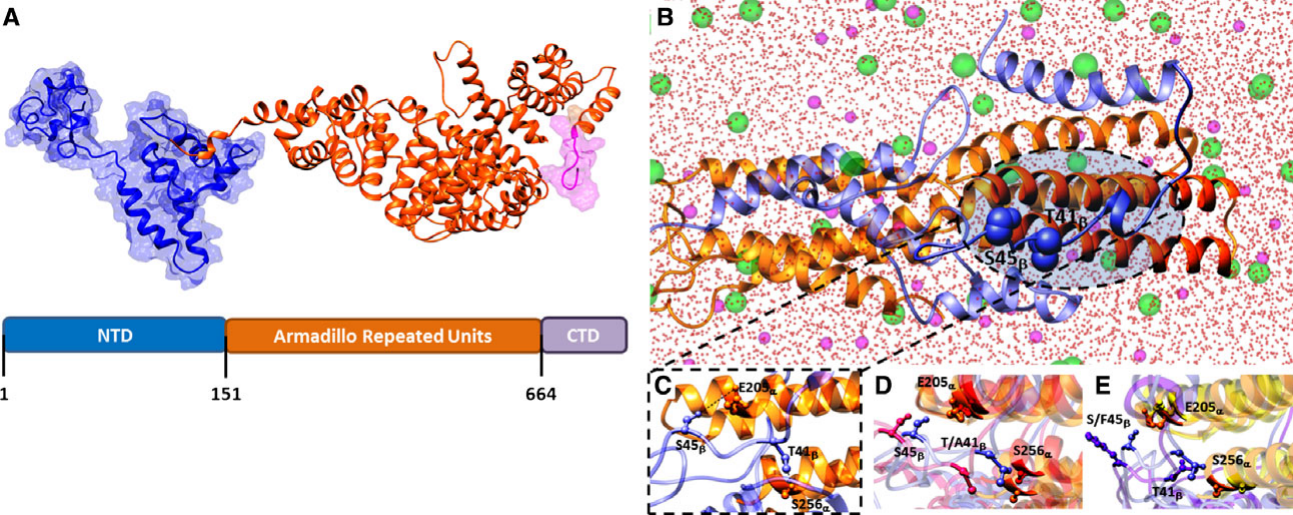
Wild‐type (WT) and mutated β‐catenins have different thermodynamic stability and affinity for α‐catenin. (A) Three‐dimensional structure of the β‐catenin WT protein. The different domains of β‐catenin are shown in different colors (same for 3D model and diagram): N‐terminal domain (NTD), blue; Armadillo repeated units, orange; C‐terminal domain (CTD), purple. (B) Overall and (C) zoomed view of an equilibrated MD snapshot of the WT β‐catenin/α‐catenin complex. The proteins are visualized in ribbon style and colored as follows: α‐catenin, cornflower blue; β‐catenin, orange. Oxygen water atoms are represented as red spheres, while chlorine and sodium are depicted as green and purple spheres, respectively. (D) Superposition of MD‐equilibrated snapshots of WT (cornflower blue) and the T41A (orange)‐mutant β‐catenin in complex with α‐catenin. (E) Superposition of MD‐equilibrated snapshots of WT (cornflower blue) and the S45F (purple)‐mutant β‐catenin in complex with α‐catenin.

The N‐terminal domain of β‐catenin, which contains the residues T41 and S45, mediates protein degradation and interacts with α‐catenin in the cell–cell adhesions mechanically coupling the actin cytoskeletons of adjacent cells (Jiang and Struhl, 1998; Pokutta *et al*., 2014). In this scenario, we performed extensive MD simulations to investigate possible effects exerted by the two mutations T41A and S45F on the protein thermodynamic stability and on β‐catenin/α‐catenin dimer formation compared to β‐catenin WT. To the purpose, the 3D model of the full‐length β‐catenin (Fig. 1A) was built starting from the available crystallographic structures and linking the missing loops previously modeled by computational techniques.

Computer‐based simulations revealed that the thermodynamic stabilities (i.e., Gibbs free energy ΔG) of the two mutant T41A and S45F β‐catenin isoforms are comparable (ΔG_T41A_ = −10 480.40 ± 0. 22 kcal·mol^−1^, ΔG_S45F_ = −10 480.33 ± 0.25 kcal·mol^−1^); rather, both mutant proteins showed a slightly increased stability compared to the WT counterpart, the T41A and S45F being more stable than the WT β‐catenin by 1.33 kcal·mol^−1^ and 1.26 kcal·mol^−1^, respectively.

Concerning β‐catenin/α‐catenin complex formation, computer simulations showed that protein/protein interface involves the N‐terminal domain of β‐catenin and two α‐helices of α‐catenin spanning residues P200‐R229 and A234‐Q259, respectively (Fig. 1B). We also noted that residues T41 and S45 of β‐catenin play an active role in the complex formation, being involved in hydrogen bonds with the side chain of S256 and E205 α‐catenin residues, respectively (Fig. 1C). The free energy of interaction (ΔG_bind_) between α‐ and WT β‐catenin is shown in the first row of Table S1. In addition, in the WT β‐catenin, both hydrogen bonds connecting the two protein chains (through T41 on the β‐chain and S256 on the α‐chain, and S45 on the β‐chain and E205 on the α‐chain, respectively) were stable and characterized by an optimal average dynamic length (ADL) of 1.95 ± 0.03 and 1.96 ± 0.04 Å, respectively. The same simulation procedure applied to α‐catenin in complex with the two mutant β‐catenin chains showed that the affinity of both mutant isoforms for α‐catenin is strongly reduced compared to the WT protein (by 2.74 and 2.87 kcal·mol^−1^, respectively), ultimately resulting in the formation of a weaker complex (Table S1).

The presence of either missense substitutions (A and F at positions 41 and 45 of β‐catenin, respectively) results in a plummet of the stabilizing interactions of the corresponding α‐/β‐catenin complexes. Indeed, the substitution of two polar residues, both featuring an –OH group available for hydrogen binding, with two residues devoid of such feature led to global conformational changes, which involve protein regions at a distal position from the mutant loci (Fig. 1D,E). As further indication of the detrimental effect exerted by the two mutations on the α‐/β‐catenin complex formation, we detected that the residual hydrogen bonds (i.e., the one between T41 on the β‐catenin and S256 on α‐catenin and between S45 on β‐catenin and E205 on α‐catenin, respectively) are longer, and hence, weaker, than in the case of the WT β‐catenin/α‐catenin complex (ADL_(T41_β_‐S256_α_)_ = 2.15 ± 0.04 Å for the T41A‐mutant and ADL_(S45_β_‐E205_α_)_ = 2.14 ± 0.06 Å for the S45F‐mutant complexes).

Overall, both mutant proteins were more stable than the WT β‐catenin and formed a weaker complex with α‐catenin due to longer hydrogen bonds.

### 3.2 *CTNNB1*‐mutated and WT DFs have different gene expression profiles

**Figure 2:**
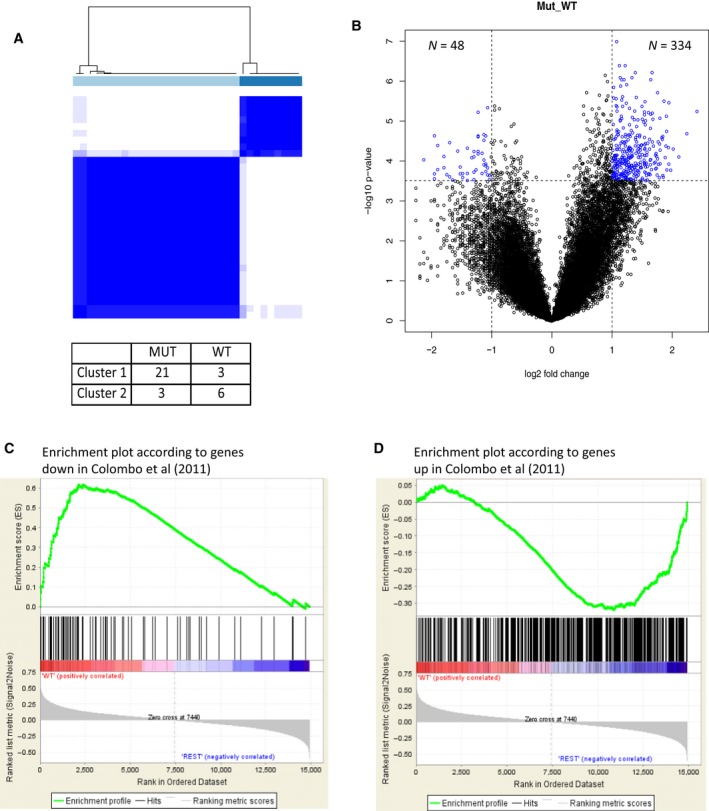
Mutated and wild‐type (WT) β‐catenin desmoid‐type fibromatosis (DFs) have different gene expression profiles. (A) Upper panel: consensus unsupervised clustering analysis of the gene expression of 33 DF samples (nine WT and 24 mutated); the heatmap depicts the consensus matrix imposing the presence of two clusters on the dataset. The values range from 0 (white, samples do not cluster together) to 1 (blue, samples showing high clustering affinity); lower panel: distribution of WT and mutated DF samples in the two clusters. (B) Volcano plot of log_2_ (fold changes) versus −log_10_ *P*‐value. The volcano plot shows transcriptional differences between the two groups of samples identified by consensus unsupervised clustering analysis. The dashed lines denote the FDR < 0.01 cutoff. (C, D) The list of genes, down (119) and up (636), in sporadic DFs (*n* = 14) as compared to normal samples/solitary fibrous tumors (*n* = 11) (31), when used as custom gene set in GSEA, identified in WT *CTNNB1* cases (panel C) a significant enrichment of genes present in normal/solitary fibrous tissues, and in mutated *CTNNB1* cases (panel D) a significant enrichment of genes present in sporadic DF.

We analyzed the gene expression profile of 33 primary surgical DFs entering our case material through DASL whole‐genome microarrays, resulting in the detection of 20 284 probe sets corresponding to 14 664 unique genes. In order to explore the inherent gene expression patterns present in our dataset, consensus unsupervised clustering was applied and revealed the presence of two main clusters of DF samples (Fig. 2A upper panel), essentially reflecting *CTNNB1*‐mutated cases (Cluster 1: 21/24 samples are mutated) and WT (Cluster 2: 6/9 samples are WT) (*P *=* *0.0047 by χ^2^, Fig. 2A lower panel). By supervised analysis and class comparison, we identified 382 differentially expressed probe sets (See Table S2 for details). The probability of finding 382 probes significant by chance if there were no real differences between the classes was 0.003, as determined by the global test. As depicted in Fig. 2B, 48 probes are upregulated in WT, while 334 are upregulated in *CTNNB1*‐mutated DFs. The gene signatures derived from Colombo *et al*. (2011) comparing sporadic DFs (*n* = 14) and normal samples/solitary fibrous tumor specimens (*n* = 11) were applied to our dataset as custom gene sets in GSEA; this analysis identified in WT β‐catenin cases a significant enrichment of genes present in normal tissues (Fig. 2C) and in mutated cases a significant enrichment of genes present in sporadic DFs (Fig. 2D).

**Figure 3:**
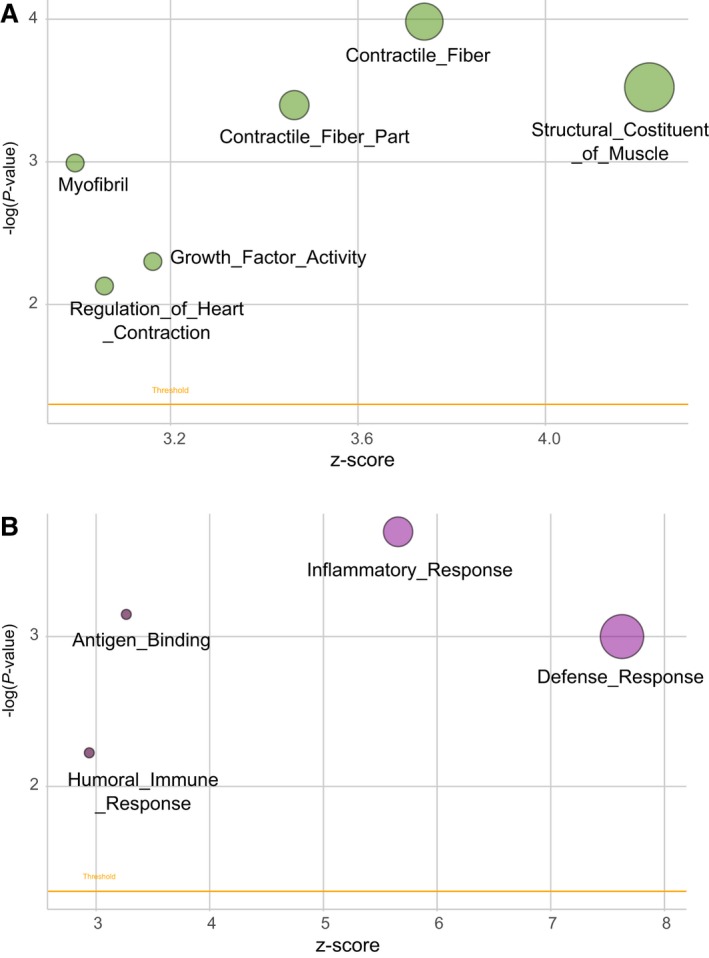
Biological pathways modulated by *CTNNB1* mutational status. Bubble plot of gene sets significantly enriched in: (panel A) *CTNNB1*‐mutated DF as compared to WT DF; (panel B) *CTNNB1* T41A DFs as compared to S45F. An overview of GSEA‐enriched networks is depicted. The *x*‐axis indicates the *z*‐score for each term, while the *y*‐axis is the negative logarithm of the adjusted *P*‐value. The area of the displayed circles is proportional to the number of genes assigned to each term. DF, desmoid‐type fibromatosis; WT, wild‐type.

To gain further insight into the biological pathways modulated by *CTNNB1* mutational status, GSEA analysis was performed. This analysis revealed six gene sets significantly enriched in *CTNNB1*‐mutated DF as compared to WT DFs. The main difference between the WT and mutated tumors was associated with their contractile fiber status, being the WT DFs characterized by an enrichment of the genes sets ‘Contractile Fiber’, ‘Structural Constituent Muscle’, and ‘Myofibril’ (Fig. 3A). This is in line with the characteristics of DF in general where myofibroblasts are dismounted and collagen is increased.

### 3.3 T41A‐mutated DFs have an upregulation of gene sets belonging to inflammatory status

No differentially expressed genes were observed when the two specific mutations T41A and S45F were compared. However, GSEA revealed four gene sets significantly enriched in *CTNNB1* T41A DFs when compared with S45F. The main difference between the two mutated tumor groups was associated with their inflammatory status, being the T41A DFs characterized by upregulation of genes of ‘Inflammatory Response’, ‘Defense Response’, ‘Humoral Immune Response’, and **‘**Antigen Binding’ (Fig. 3B). In each of these four gene sets, the upregulated genes included mainly chemokine ligands and receptors and interleukins. This intriguing evidence suggested that the inflammation and immune response might play a role particularly in T41A cases.

### 3.4 T41A‐ and S45F‐mutated DFs have different expression of inflammation‐related genes

On the basis of the inflammation‐related gene set enrichment found in T41A cases, we further explored by nanostring the expression of 249 inflammation‐related biomarkers including chemokines, interleukins, growth factors, tool‐like receptors.

Comparing both mutated (T41A + S45F) versus WT DFs, among the 139 biomarkers resulted to be expressed in all the 33 samples, only HMGN1 was found statistically significantly overexpressed in mutated DFs (adjusted FDR *P*‐value = 0.009). This nucleosome‐binding protein HMGN1 is a potent alarmin that promotes T cell‐mediated antitumor immunity (Wei *et al*., 2014).

Further analysis comparing the two mutated groups demonstrated that 147 biomarkers were expressed in all the 24 samples; 16 of them were statistically significantly deregulated in the T41A compared with S45F cases (Table**** 2). In detail, 14 genes resulted to be overexpressed in T41A DFs (CREB1, MAPK3, CCL4, TLR2, HMGN1, MKNK1, MEF2A, IRF1, CD55, PPP1R12B, RAPGEF2, C2, PTGER2, TLR3) and TGF‐β2 and TGF‐β3 were downregulated.

**Table 2:** Nanostring analysis comparison between T41A‐ and S45F‐mutated desmoid‐type fibromatosis

Gene	Fold change	*P*‐value	Adjusted *P*‐value
CREB1	1.3269	0.00001	0.0010
MAPK3	1.2038	0.00005	0.0037
CCL4	3.2859	0.00037	0.0168
TGFB2	0.5706	0.00055	0.0168
TLR2	1.4818	0.00066	0.0168
HMGN1	1.2573	0.00081	0.0168
MKNK1	1.2910	0.00087	0.0168
TGFB3	0.6707	0.00092	0.0168
MEF2	1.2359	0.00149	0.0242
IRF1	1.6080	0.0017	0.0242
CD55	1.6027	0.00183	0.0242
PPP1R12B	1.6331	0.00198	0.0242
RAPGEF2	1.1710	0.00312	0.0353
C2	1.6592	0.00472	0.0495
PTGER2	1.6147	0.00522	0.0498
TLR3	1.9329	0.00542	0.0498

Interestingly, some of these upregulated markers are known to be associated with antitumor immunity, such as HMGN1 (Wei *et al*., 2014); the transcription factor CREB1 that plays a key role in NF‐kB inhibition, thereby limiting proinflammatory responses and enhancing host immune responses (Wen *et al*., 2010); IRF1 that contributes to tumor immune surveillance (Dou *et al*., 2014). Moreover, the lower levels of TGF‐β2 and TGF‐β3 we observed in T41A are intriguing due to the bifunctional role (pro‐ and anti‐inflammatory) of these two cytokines recently described in immune system (Okamura *et al*., 2015).

Cumulatively, the main differences dictated by the RNA analyses were the augment of secreted proteins enhancing tumor immune response of T41A compared to S45F mutation.

### 3.5 Host immunoscore system assessment in T41A‐ and S45F‐mutated DFs

**Figure 4:**
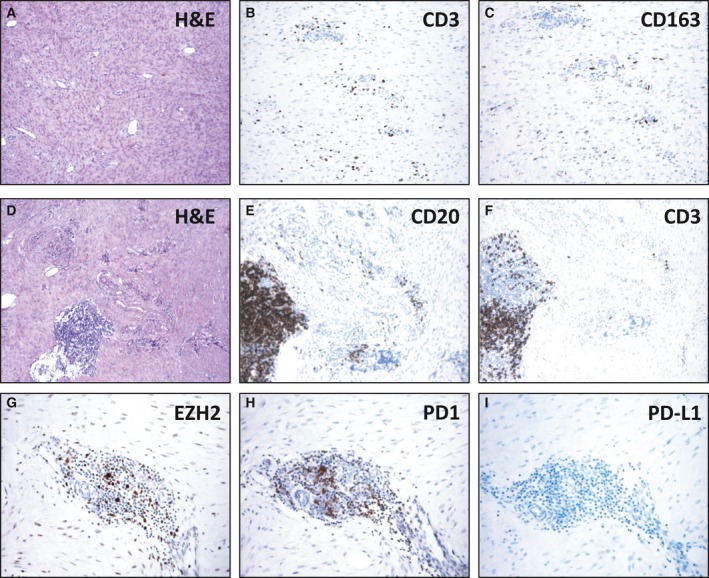
T cells and tumor‐associated macrophages (TAM) are rare in T41A‐ or S45F‐mutated desmoid‐type fibromatosis. In the tumor proliferation (A), a low number of CD3^+^ T cells (B) and CD163^+^ tumor‐associated macrophages (C) were distributed around the vessels. In occasional small lymphoid aggregates (D), CD20^+^ B cells (E), CD3^+^ T cells (F), and EZH2^+^ cells (G) were present. Few PD‐1‐decorated lymphocytes were restricted to lymphoid aggregates (H), and no immunolabeled PD‐L1 tumor‐associated inflammatory cell (I) was present.

We made immunoscore assessment of the immunosystem‐based biomarkers including CD3, CD4, CD8, FOXP3, CD20, CD56, CD21, CD163, and CD68. In terms of immunoprofile between the two types of mutations, no difference was observed. In all cases, the analysis revealed throughout the tumor proliferation a low number of T cells, CD3^+^, and CD8^+^ and of tumor‐associated macrophages (TAM), CD163^+^, and/or CD68^+^, distributed around the vessels (Fig. 4A–C). In addition, a more consistent number of tumor‐infiltrating lymphocytes (TILs), T cells, and/or TAMs were present at the boundaries, where the proliferation abutted the skeletal muscle, or in the area of entrapped muscle fibers which in all the cases exhibited marked regressive changes (Fig. 4D,E). No FOXP3‐, CD4‐, and CD56‐immunolabeled cells were observed. In addition to T cells, CD20^+^ B cells (Fig. 4F) were present in occasional small lymphoid aggregates that did not reach characteristics of tertiary lymphoid structures lacking CD21‐decorated dendritic follicular cells and/or EZH2‐positive (here applied as surrogate of activation) germinative centers (Fig. 4G) (Neyt *et al*., 2012).

Despite the lack of a sizable number of EZH2 expressing T lymphocytes, we explored the expression of the costimulatory molecule PD‐1 and its ligand PD‐L1 in order to identify potential treatment window of the host immune system being, as expected, tumor cells always negative. The analysis, performed on nine cases, showed only few PD‐1‐decorated lymphocytes, mostly restricted to lymphoid aggregates (Fig. 4H), and no immunolabeled PD‐L1 tumor‐associated inflammatory cells (Fig. 4I).

Altogether, the host cellular immunocomponent was very poor, suggesting that the observed modulation of the secreted cytokines/chemokines was sustained by tumor cellular component other than the resident TILs and TAMs. By contrast, in all mutated and WT cases, all the tumor cells were β‐catenin‐immunodecorated.

## 4 Discussion

We started from the hypothesis that *CTNNB1* mutation types can influence the β‐catenin stability and its affinity for α‐catenin, besides the pattern of gene expression and consequently DF behavior.

The modeling results demonstrated that the presence of T41A or S45F mutation concurs in the stabilization of the mutated proteins compared to the WT, and in the reduction in the affinity binding with α‐catenin. The *in silico* results supported the assumption that both mutations shift the balance between membrane and cytoplasmic β‐catenin toward a cytoplasmic/nuclear pool, as showed by the nuclear and cytoplasmic immunoreactivity of β‐catenin in mutated DF (Signoroni *et al*., 2007).

Next, we investigated by gene expression analysis the influence of the two mutations on β‐catenin nuclear role as transcription factor. This analysis revealed that the group of T41A + S45F showed a distinct signature compared to WT DFs, suggesting that they are two distinct biological entities. Interestingly, the two different patterns of gene expression are in line with the clinical evidence that β‐catenin WT DFs are associated with a better prognosis than mutated ones (van Broekhoven *et al*., 2015; Colombo *et al*., 2013; Dômont *et al*., 2010).

Our profiling analysis is quite unique. Indeed, only a recent gene expression study compared mutated versus WT DFs, indicating that mutated DFs did not cluster separately and did not have a different clinical behavior from WT tumors (Crago *et al*., 2015). However, the authors of this study included in the ‘mutated’ group also tumors lacking *CTNNB1* mutation but carrying other Wnt/β‐catenin signaling alterations, such as *APC* mutations and/or loss. Thus, their results – rather than elucidating possible differences between *CTNNB1‐*mutated and WT DFs – reflect the rarity of the ‘wild‐type’ Wnt/β‐catenin genotype. This prompted us to explore *APC* status in our series by next‐generation sequencing that revealed the absence of *APC* mutations in all but one *CTNNB1* WT case (data not shown).

The other gene expression analyses on DFs reported in the literature are not comparable with our study because, regardless of tumor *CTNNB1* mutational status, they compared DFs with nodular fasciitis, normal tissues, and/or solitary fibrous tumors in order to find specific gene signatures associated with DF biology or outcome (Bacac *et al*., 2006; Colombo *et al*., 2011; Denys *et al*., 2004; Salas *et al*., 2010; Skubitz and Skubitz, 2004). However, it should be mentioned that one of these studies comparing DFs with normal tissues identified two distinct subgroups of DFs on the basis of gene expression profiles that paralleled different clinical outcomes (Skubitz and Skubitz, 2004). Interestingly, the subsequent sequencing of the same samples revealed that these two subclusters showed different β‐catenin mutation distribution (Misemer *et al*., 2014), even if the exiguous number of analyzed cases did not allow drawing any definitive conclusion.

When the gene signatures previously obtained comparing sporadic DFs and normal/solitary fibrous samples were applied to our dataset as custom gene sets in GSEA, we found a significant enrichment of genes described in normal tissues in the group of WT β‐catenin DFs, and an enrichment of genes present in sporadic DFs in the mutated cases (Colombo *et al*., 2011). This evidence suggests that WT DFs share molecular characteristics with normal tissue that may explain their better prognosis than mutated DFs.

No differently expressed genes were observed when the two specific mutations T41A and S45F were compared, supporting that the two specific mutations dictate a similar transcriptional activity of the two corresponding mutant β‐catenin proteins. However, when the comparison between T41A and S45F was made using gene ontology enrichment analysis, a difference in specific pathways belonging to ‘Inflammatory Response’, ‘Defense Response’, ‘Humoral Immune Response’, and ‘Antigen Binding’ emerged. As DFs have unpredictable clinical course, an equilibrium between host and disease may be crucial. To further investigate a possible role of immune system/inflammation in DFs, the expression of human inflammation‐related biomarkers was assessed by Nanostring. According to gene ontology enrichment analysis, the comparison between the two mutated DFs revealed 16 genes statistically significant up‐ or downregulated in T41A compared with S45F DFs. Due to the known relationship between oncogene activation and inflammation according to which oncogenes can also regulate the inflammatory milieu in tumors (Dibra *et al*., 2014), we can speculate that by activating specific pro‐ and anti‐inflammatory mediators, the two *CTNNB1* mutations may produce a different inflammatory milieu that can have different impact on DF behavior. Coherently, T41A cases, generally associated with better prognosis after surgery, showed overexpression of anti‐inflammatory markers associated with antitumor immunity, such as CREB1, HMGN1, MKNK1 and IRF1, as well as lower levels of TGF‐β3 and TGF‐β2 associated with proinflammatory activities. We believe that these biomarkers were mainly derived from tumor cells because throughout all tumor proliferations, from which RNA has been extracted for gene expression, only a low number of TILs and TAMs were observed and a more consistent number of TILs, T cells, and/or TAMs were all restricted to the boundaries of DFs, where the proliferation abutted the skeletal muscle or in the area of entrapped muscle fibers. This peculiar inflammatory microenvironment is in line with the hypothesis that β‐catenin signaling activation, from which DFs depend, results in T‐cell exclusion favoring the immune evasion. Indeed, by inhibiting T‐cell activation, β‐catenin activation is one of the mechanisms that melanoma can use to evade antitumor immunity (Spranger *et al*., 2015). Here, the PD1/PD‐L1 profile of DFs fell into the group defined ‘the noninflamed tumor type’, which is closely associated with the EMT/STEM‐like type controlled by oncogenic and epigenetic pathways including β‐catenin, and is predictive of no clinical response of DFs to immune‐based therapies (Zou *et al*., 2016). Rather, the pharmacological inhibition of β‐catenin activity for tumor therapy has come into the focus of drug development (Käfer *et al*., 2016). Moreover, the difference in the inflammatory milieu between the two mutated forms T41A and S45F deserves further study as it can be useful in guiding future tailored therapies.

In conclusion, our study demonstrated that DFs carrying T41A or S45F mutations and WT β‐catenin are two distinct molecular subgroups with regard to β‐catenin stability, α‐catenin affinity, and gene expression profiling. Particularly, WT DFs showed an expression profile more similar to normal tissues, in keeping with their better prognosis. Moreover, we provided evidence that a different inflammation signature characterized S45F‐ and T41A‐mutated cases, suggesting a specific inflammation setting mediated by the type of β‐catenin mutation. Finally, all mutated cases showed a low number of TIL and TAM cells and a low or absent expression of PD‐1 and PD‐L1, consistent with β‐catenin activation insensitive to checkpoint blockade. Considering the limitations of the study (small sample size, single‐center experience), further studies are needed to confirm our data.

## Author contributions

CC, FP, Sabrina Pricl, and Silvana Pilotti involved in conception and design; CC, AB, NP, LD, EL, MF, and SB acquired data; FP, Sabrina Pricl, SC, and PV analyzed and interpreted the data; AG, Silvana Pilotti, SS, MF, and EP participated in revising the article. All the authors gave final approval of the version to be submitted.

## Supporting information


**Table S1.** Binding free energies (ΔG_bind_) and binding free energy differences (ΔΔG_bind_) for the WT, T41A and S45F β‐catenin in complex with α‐catenin.

**Table S2.** List of the 382 genes differentially expressed between mutated‐ and wt‐DT patients.

**Data S1.** Computational details.
