# Supplementary material for: β‐Catenin in desmoid‐type fibromatosis: deep insights into the role of T41A and S45F mutations on protein structure and gene expression
Source: Mol Oncol. 2017 Sep 29;11(11):1495–507. doi: 10.1002/1878-0261.12101 (PMC5664003; doi:10.1002/1878-0261.12101)
Supplement: Supplementary file 1 — Table S1. Binding free energies (ΔGbind) and binding free energy differences (ΔΔGbind) for the WT, T41A and S45F β‐catenin in complex with α‐catenin. [file MOL2-11-1495-s001.docx]

|  | ΔG_bind_  _(kcal/mol)_ | ΔΔG_bind_  _(kcal/mol)_ |
| --- | --- | --- |
| WT | -13.15 ± 0.26 | - |
| T41A | -10.41 ± 0.28 | -2.74 |
| S45F | -10.28 ± 0.28 | -2.87 |

**Supplementary Table 1**. Binding free energies (ΔG_bind_) and binding free energy differences (ΔΔG_bind_) for the WT, T41A and S45F β-catenin in complex with α-catenin.

According to its definition (ΔΔG_bind_=ΔG_bind(WT)_-ΔG_bind(MUT)_), negative values of ΔΔG_bind_ indicate that the considered amino acid substitution at a given position of β-catenin is unfavorable in terms of interaction with α-catenin.
