# Supplementary material for: β‐Catenin in desmoid‐type fibromatosis: deep insights into the role of T41A and S45F mutations on protein structure and gene expression
Source: Mol Oncol. 2017 Sep 29;11(11):1495–507. doi: 10.1002/1878-0261.12101 (PMC5664003; doi:10.1002/1878-0261.12101)
Supplement: Supplementary file 3 — Data S1. Computational details. [file MOL2-11-1495-s003.docx]

**Computational details**

The Amber ff14SB force field (1) was used to parameterize all protein structures. All simulations were carried out using the *Pmemd* modules of Amber 14 (2), running on a hybrid CPU/GPU calculation cluster.

The optimized full-length three-dimensional (3D) models of wt and mutant isoforms of β-catenin were obtained by a combination of homology modeling techniques and molecular dynamics refinements. The protein model was built starting from the available structures of the phosphorylation motif (aa. Lys19-Pro44, 2G57.pdb (3); aa. Asp83-Gln143, 4ONS.pdb (4)), and of the armadillo repeats domain with part of the protein C-terminal (2Z6H.pdb (5)). The models of the T41A and S45F variant proteins were obtained by mutating the relevant amino acids, and optimizing the corresponding isoforms via energy relaxation followed by MD simulations in solution in agreement with a well-validated computational procedure (6).

The α-catenin/β-catenin protein-protein interface and the docking modes of α-catenin (4IGG.pdb, (7)) onto the β-catenin WT and its two truncated isoforms were determined using the HADDOCK server (8). The resulting protein/protein docked conformations were clustered and visualized; then, the structure of each complex characterized by the lowest interaction energy in the prevailing cluster was selected for further modeling (6). The selected α-catenin/β-catenin complexes were then solvated in a TIP3P (9) water box and, then, the required amount of Na^+^ and Cl^-^ ions were added to neutralize the system and to mimic physiological salt conditions (150 mM), removing eventual overlapping water molecules. The solvated systems were subjected to a combination of steepest descent/conjugate gradient minimization of the potential energy, during which all bad contacts were relieved. The relaxed systems were then gradually heated to 300 K in three intervals by running constant volume-constant temperature (NVT) MD simulation, allowing a 0.5 ns interval per 100 K. Subsequently, 40 ns MD simulations under isobaric-isothermal (NPT) conditions were conducted to fully equilibrate each solvated compound. The *SHAKE* algorithm (10) with a geometric tolerance of 5x10^-4^ Å was imposed on all covalent bonds involving hydrogen atoms. Temperature control was achieved using the Langevin temperature equilibration scheme and an integration time step of 2 fs. The particle mesh Ewald (PME) method (11) was used to treat the long-range electrostatics. At this point, these MD runs were followed by other 200 ns of NVT MD simulation. The last 100 ns of the MD data collection period described were used for the calculation of the binding free energy between the two proteins via the MM/PBSA (Molecular Mechanincs/Poisson Boltzmann Surface Area) approach (6).

According to this theory, the free energy of binding (ΔG_bind_) between α-catenin and each β-catenin variant can be calculated as the sum of different energetic contributions, corresponding to the average MD energies (ΔE_MM_ = ΔE_ele_ + ΔE_vdW_), the average solvation free energy (ΔG_solv_ = ΔG_solv,pol_ + ΔG_solv,nonpol_), and the entropic contribution (−TΔS).

**Table SI1**. Binding free energies (ΔG_bind_) and binding free energy differences (ΔΔG_bind_) for the wt, T41A and S45F β-catenin in complex with α-catenin.

|  | ΔG_bind_ (kcal/mol) | ΔΔG_bind_ (kcal/mol) |
| --- | --- | --- |
| WT | -13.15 ± 0.26 | - |
| T41A | -10.41 ± 0.28 | -2.74 |
| S45F | -10.28 ± 0.28 | -2.87 |

**References**

1. Maier JA, Martinez C, Kasavajhala K, Wickstrom L, Hauser KE, Simmerling C. ff14SB: improving the accuracy of protein side chain and backbone parameters from ff99SB. J Chem Theory Comput. 2015;11(8): 3696-13.

2. Case DA, Babin, V, Berryman JT, Betz RM, Cai Q, Cerutti DS, et al. AMBER 14, University of California, San Francisco, CA, USA, 2014.

3. Megy S, Bertho G, Gharbi-Benarous J, Baleux F, Benarous R, Girault JP. STD and TRNOESY NMR studies for the epitope mapping of the phosphorylation motif of the oncogenic protein beta-catenin recognized by a selective monoclonal antibody. Febs Lett. 2006;580:5411-22.

4. Pokutta S, Choi HJ, Ahlsen G, Hansen SD, Weis WI. Structural and thermodynamic characterization of cadherin·β-catenin·α-catenin complex formation. J Biol Chem. 2014;289(19):13589-601.

5. Xing Y, Takemaru K, Liu J, Berndt JD, Zheng JJ, Moon RT, Xu W. Crystal structure of a full-length beta-catenin. Structure. 2008;16(3):478-87.

(6) a) Morgan A, Gandin I, Belcaro C, Palumbo P, Palumbo O, Biamino E, Dal Col V, Laurini E, Pricl S, Bosco P, Carella M, Ferrero GB, Romano C, d'Adamo AP, Faletra F, Vozzi D. Target sequencing approach intended to discover new mutations in non-syndromic intellectual disability. Mutat Res. 2015;781:32-6; b) Brambilla L, Genini D, Laurini E, Merulla J, Perez L, Fermeglia M, Carbone GM, Pricl S, Catapano CV. Hitting the right spot: Mechanism of action of OPB-31121, a novel and potent inhibitor of the Signal Transducer and Activator of Transcription 3 (STAT3). Mol Oncol. 2015;9(6):1194-206; c) Pricl S, Cortelazzi B, Dal Col V, Marson D, Laurini E, Fermeglia M, Licitra L, Pilotti S, Bossi P, Perrone F. Smoothened (SMO) receptor mutations dictate resistance to vismodegib in basal cell carcinoma. Mol Oncol. 2015;9(2):389-97; d) Brune S, Schepmann D, Klempnauer KH, Marson D, Dal Col V, Laurini E, Fermeglia M, Wünsch B, Pricl S. The sigma enigma: in vitro/in silico site-directed mutagenesis studies unveil σ1 receptor ligand binding. Biochemistry. 2014;53(18):2993-3003; e) Gibbons DL, Pricl S, Posocco P, Laurini E, Fermeglia M, Sun H, Talpaz M, Donato N, Quintás-Cardama A. Molecular dynamics reveal BCR-ABL1 polymutants as a unique mechanism of resistance to PAN-BCR-ABL1 kinase inhibitor therapy. Proc Natl Acad Sci U S A. 2014;111(9):3550-5; f) Bozzi F, Conca E, Laurini E, Posocco P, Lo Sardo A, Jocollè G, Sanfilippo R, Gronchi A, Perrone F, Tamborini E, Pelosi G, Pierotti MA, Maestro R, Pricl S, Pilotti S. In vitro and in silico studies of MDM2/MDMX isoforms predict Nutlin-3A sensitivity in well/de-differentiated liposarcomas. Lab Invest. 2013;93(11):1232-40; g) Conca E, Miranda C, Dal Col V, Fumagalli E, Pelosi G, Mazzoni M, Fermeglia M, Laurini E, Pierotti MA, Pilotti S, Greco A, Pricl S, Tamborini E. Are two better than one? A novel double-mutant KIT in GIST that responds to Imatinib. Mol Oncol. 2013;7(4):756-62; h) Gibbons DL, Pricl S, Kantarjian H, Cortes J, Quintás-Cardama A. The rise and fall of gatekeeper mutations? The BCR-ABL1 T315I paradigm. Cancer. 2012;118(2):293-9; i) Pierotti MA, Tamborini E, Negri T, Pricl S, Pilotti S. Targeted therapy in GIST: in silico modeling for prediction of resistance. Nat Rev Clin Oncol. 2011;8(3):161-70; j) Dileo P, Pricl S, Tamborini E, Negri T, Stacchiotti S, Gronchi A, Posocco P, Laurini E, Coco P, Fumagalli E, Casali PG, Pilotti S. Imatinib response in two GIST patients carrying two hitherto functionally uncharacterized PDGFRA mutations: an imaging, biochemical and molecular modeling study. Int J Cancer. 2011;128(4):983-90; k) Carta A, Pricl S, Piras S, Fermeglia M, La Colla P, Loddo R. Activity and molecular modeling of a new small molecule active against NNRTI-resistant HIV-1 mutants. Eur J Med Chem. 2009;44(12):5117-22; l) Conca E, Negri T, Gronchi A, Fumagalli E, Tamborini E, Pavan GM, Fermeglia M, Pierotti MA, Pricl S, Pilotti S. Activate and resist: L576P-KIT in GIST. Mol Cancer Ther. 2009;8(9):2491-5; m) Woodman SE, Trent JC, Stemke-Hale K, Lazar AJ, Pricl S, Pavan GM, Fermeglia M, Gopal YN, Yang D, Podoloff DA, Ivan D, Kim KB, Papadopoulos N, Hwu P, Mills GB, Davies MA; n) McAuliffe JC, Wang WL, Pavan GM, Pricl S, Yang D, Chen SS, Lazar AJ, Pollock RE, Trent JC. Unlucky number 13? Differential effects of KIT exon 13 mutation in gastrointestinal stromal tumors. Mol Oncol. 2008;2(2):161-3; o) Negri T, Pavan GM, Virdis E, Greco A, Fermeglia M, Sandri M, Pricl S, Pierotti MA, Pilotti S, Tamborini E. T670X KIT mutations in gastrointestinal stromal tumors: making sense of missense. J Natl Cancer Inst. 2009;101(3):194-204; p) Ferrone M, Perrone F, Tamborini E, Paneni MS, Fermeglia M, Suardi S, Pastore E, Delia D, Pierotti MA, Pricl S, Pilotti S. Functional analysis and molecular modeling show a preserved wild-type activity of p53(C238Y). Mol Cancer Ther. 2006;5(6):1467-73; q) Tamborini E, Pricl S, Negri T, Lagonigro MS, Miselli F, Greco A, Gronchi A, Casali PG, Ferrone M, Fermeglia M, Carbone A, Pierotti MA, Pilotti S. Functional analyses and molecular modeling of two c-Kit mutations responsible for imatinib secondary resistance in GIST patients. Oncogene. 2006;25(45):6140-6; r) Pricl S, Fermeglia M, Ferrone M, Tamborini E. T315I-mutated Bcr-Abl in chronic myeloid leukemia and imatinib: insights from a computational study. Mol Cancer Ther. 2005;4(8):1167-74.

7. Rangarajan ES, Izard T. Dimer asymmetry defines α-catenin interactions. Nat Struct Mol Biol. 2013;20(2):188-93.

8. Dominguez C, Boelens R, Bonvin AMJJ. HADDOCK: a protein-protein docking approach based on biochemical or biophysical information. J Am Chem Soc. 2003;125:1731-7.

9. Jorgensen WL, Chandrasekhar J, Madura JD, Impey RW, Klein ML. Comparison of simple potential functions for simulating liquid water. J Chem Phys. 1983;79:926-35.

10. Ryckaert JP, Ciccotti G, Berendsen HJC. Numerical integration of the Cartesian equations of motion of a system with constraints: molecular dynamics of n-alkanes.

11. Toukmaji A, Sagui C, Board J, Darden T. Efficient particle-mesh Ewald based approach to fixed and induced dipolar interactions. J Chem Phys 2000;113:10913-27.
